# Multiple Trocar-Site Hernias Following Robot-Assisted Incisional Hernia Repair: A Case Report

**DOI:** 10.7759/cureus.111237

**Published:** 2026-06-21

**Authors:** Zachary J Tucker, Jason Boardman

**Affiliations:** 1 General Surgery, Alabama College of Osteopathic Medicine, Dothan, USA; 2 General Surgery, AdventHealth Orlando, Orlando, USA

**Keywords:** eight mm trocar, incisional hernia, minimally invasive surgery, port site hernia, robotic surgery, trocar site hernia, trocar-site incision

## Abstract

Trocar-site hernia (TSH) is a recognized complication of minimally invasive surgery. Current guidelines suggest fascial closure for trocar sites of 10 mm or larger. However, there is limited evidence regarding the necessity of fascial closure for 8-mm trocar sites. The occurrence of multiple TSHs in a single patient following robot-assisted surgery has not been well documented. We present a rare case of three simultaneous TSHs at 8-mm robotic port sites following a robot-assisted incisional hernia repair.

A 69-year-old male with a BMI of 28.8 kg/m^2^ presented with a symptomatic umbilical trocar-site incisional hernia following prior laparoscopic inguinal hernia repair, with an associated 3-4 cm diastasis recti. He underwent a robot-assisted incisional hernia repair using the da Vinci Surgical System (Intuitive Surgical, Sunnyvale, CA, USA) via a ventral transabdominal preperitoneal approach with mesh reinforcement. Three 8-mm robotic trocars were used in the left lateral position, and fascial closure of these trocar sites was not performed. At two-week follow-up, the patient reported a new lump at the left inferior trocar site. Computed tomography confirmed a TSH containing fat in this location, which was subsequently confirmed intraoperatively to be omentum. Thirty-two days after the index operation, the patient underwent robot-assisted incisional hernia repair of this new hernia. At the time of the operation, three separate TSHs were identified at all three prior 8-mm port sites, measuring 5 mm at the left superior defect, 5 mm at the left middle defect, and 2 cm at the left inferior defect. The omentum was noted to be contained in all three sites but was incarcerated at the inferior hernia. After reduction of the omentum, the hernias were repaired with musculofascial closure and mesh reinforcement with significant overlap of more than 2 cm. The new trocar sites were closed prior to completion of the case. The patient’s subsequent recovery was uncomplicated with no evidence of recurrence.

This case demonstrates that multiple TSHs may develop at all 8-mm robotic trocar sites in a single patient, with early symptom onset and rapid progression to incarceration. A history of multiple or recurrent hernias should prompt consideration of prophylactic fascial closure of all trocar sites, regardless of trocar size. Further research is needed to establish evidence-based guidelines for fascial closure of 8-mm robotic ports and to identify patients who may benefit from prophylactic closure.

## Introduction

Trocar-site hernia (TSH) is defined as an incisional hernia occurring at the site of trocar insertion [1]. The true incidence is likely underreported, with estimates ranging from 0.1% to 16.2%, depending on the surgical procedure, method of detection, and duration of follow-up [1,2]. The higher end of this range reflects studies using routine postoperative imaging, which detect substantially more TSHs than clinical examination alone [3]. 

The pathogenesis of TSH is multifactorial, involving both patient-related and technical factors. Established patient-related risk factors include obesity, advanced age, diabetes mellitus, and pre-existing fascial defects [1,2,4,5]. Technical factors implicated in TSH development include trocar size greater than 10 mm, use of bladed trocars, midline trocar placement, prolonged operative time, and extension of the fascial incision during surgery [1,2,6]. These factors have informed current recommendations for trocar-site management, though their applicability to smaller 8-mm robotic trocar sites remains unclear. 

Since the introduction of robot-assisted surgery, 8-mm trocars have become standard across most platforms, but evidence regarding the necessity of fascial closure for these sites remains limited. Current guidelines from the European and American Hernia Societies recommend fascial closure for trocar sites of 10 mm or larger, though these recommendations do not specifically address 8-mm robotic trocars [3]. A prospective study of 166 patients undergoing robot-assisted abdominal wall surgery without fascial closure of 8-mm trocar sites reported only one TSH (0.2%) at a median follow-up of 14.5 months, suggesting that routine closure may not be necessary [7]. Similarly, studies of robot-assisted radical prostatectomy reported overall TSH rates of 0.4% in cases where only midline 12-mm ports underwent fascial closure [8]. In contrast, a long-term study with a minimum five-year follow-up reported an 18% TSH rate, with the majority occurring at midline trocar insertion sites [9]. These differences may reflect variability in procedure type, trocar location, and duration of follow-up across studies. 

Multiple simultaneous TSHs in a single patient are exceedingly rare and poorly described, particularly following robot-assisted incisional hernia repair. We present a case of three simultaneous TSHs occurring at 8-mm trocar sites within two weeks following robot-assisted incisional hernia repair. This case highlights the potential for TSH development even with smaller trocar sizes and demonstrates the importance of vigilant postoperative surveillance.

## Case presentation

A 69-year-old male presented with a painful, reducible bulge in his abdomen. His past medical history was significant for prostate cancer status post laparoscopic prostatectomy and prior laparoscopic inguinal hernia repair. His BMI was 28.8 kg/m² (weight: 88.5 kg and height: 175 cm). He was a non-smoker and rarely consumed alcohol. His medications included tadalafil 5 mg daily and a multivitamin. Preoperative laboratory values, including albumin and HbA1c, were not ordered, as there was no clinical indication.

Physical examination revealed a scaphoid abdomen without guarding or rebound tenderness. An abdominal hernia was present at the umbilical trocar site from a prior laparoscopic inguinal hernia repair. The hernia was tender and reducible, with an associated 3-4 cm diastasis.

The patient underwent robot-assisted incisional hernia repair via a ventral transabdominal preperitoneal approach using the da Vinci Surgical System (Intuitive Surgical, Sunnyvale, CA, USA) with mesh reinforcement, partial transversalis fascia release, and adjacent tissue transfer. Antibiotic prophylaxis consisted of cefazolin 3 g intravenously one hour before incision. Access was obtained in the left upper quadrant using a 5-mm Optiview trocar (Ethicon Endo-Surgery, Cincinnati, OH, USA), which was subsequently upsized to 8 mm. Two additional 8-mm trocars were placed under direct visualization in the left lateral position. Pneumoperitoneum was maintained at 15 mmHg. Intraoperatively, a reducible incisional hernia was confirmed, measuring 2 cm. A preperitoneal dissection was performed with the development of a tissue flap extending across the midline. Partial release of the transversalis fascia was required to facilitate flap advancement and tension-free closure. The midline fascial defect was closed using a Stratafix barbed suture (Ethicon, Raritan, NJ, USA) with medial advancement and reapproximation of the rectus sheaths. A 16 × 12 cm Progrip self-gripping mesh (Covidien, Mansfield, MA, USA) was placed and secured with interrupted 3-0 Vicryl (polyglactin) sutures (Ethicon, Raritan, NJ, USA). The peritoneum was closed with a V-Loc 90 barbed suture (Covidien, Mansfield, MA, USA). Estimated blood loss was minimal. The patient was discharged the same day with standard postoperative instructions, including a 20-pound lifting restriction.

At two-week follow-up, the patient reported a new lump at the left inferior trocar site. He denied fever or chills. Physical examination revealed a bulge at the left inferior trocar site that was not reducible, and computed tomography demonstrated a hernia containing fat at this trocar site, as shown in Figure 1, which was confirmed intraoperatively to be omentum. The patient was scheduled for surgical repair.

**Figure 1:**
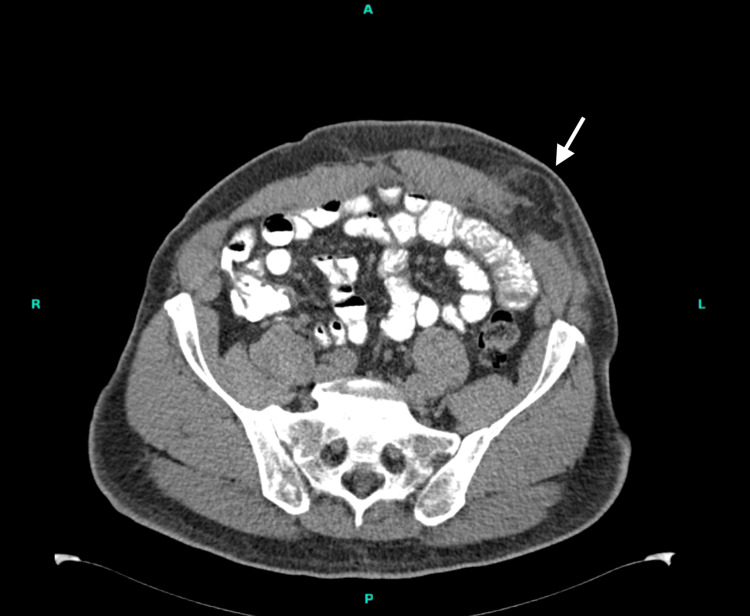
Non-contrast computed tomography of the abdomen demonstrating a defect in the anterior abdominal wall fascia at the inferior trocar site.

Fifteen days after the follow-up visit (32 days after the index operation), the patient underwent reoperation. Access for the second procedure was obtained in the right upper quadrant to avoid the prior trocar sites. Pneumoperitoneum was maintained at 15 mmHg. The total operative time was 94 minutes. Intraoperatively, three TSHs were identified at all three prior 8-mm port sites, measuring 5 mm, 5 mm, and 2 cm in size, as shown in Figures 2-4, respectively. The omentum was noted to be contained in all three sites but was incarcerated at the inferior hernia.

**Figure 2:**
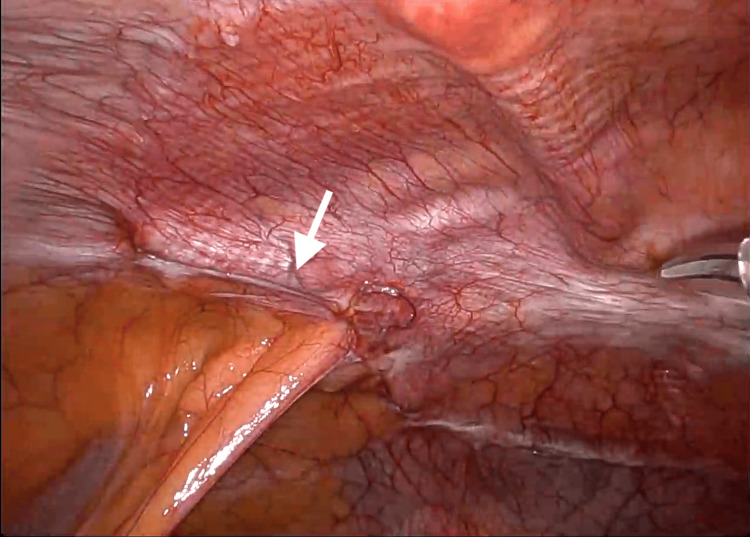
5-mm left superior trocar-site hernia

**Figure 3:**
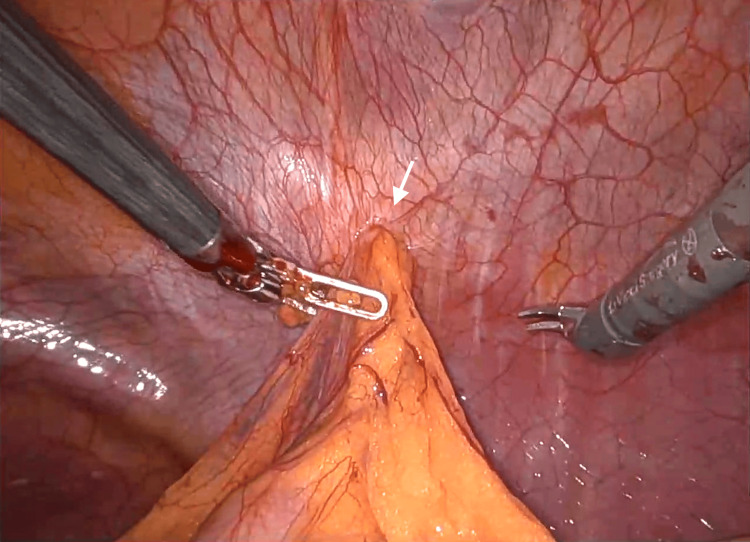
5-mm left middle trocar-site hernia

**Figure 4:**
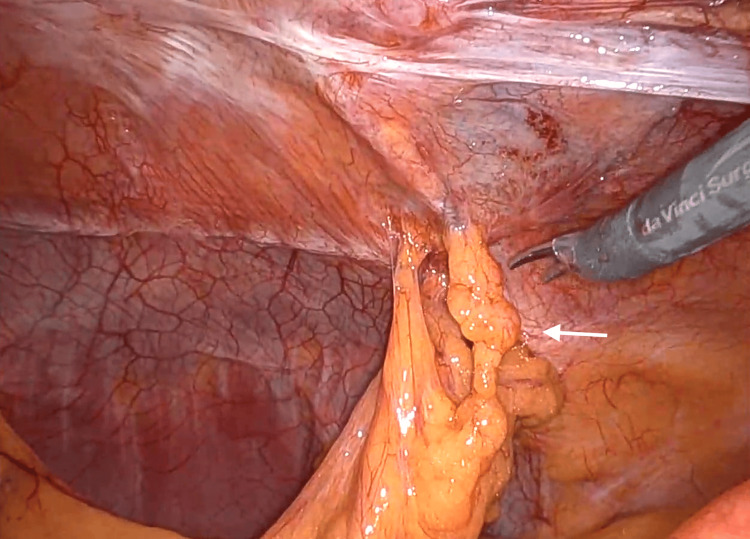
2-cm left inferior trocar-site hernia with incarcerated omentum

The superior and middle hernias were reduced robotically. Attempts to reduce the incarcerated omentum at the left inferior site were unsuccessful, and subsequent robot-assisted dissection was performed to free the hernia contents, as demonstrated in Figure 5. The prior mesh was visualized and remained intact. The preperitoneal plane was developed; however, multiple peritoneal defects were created due to the thin, friable nature of the peritoneum. Given the inability to achieve tension-free peritoneal closure, Symbotex composite mesh (Medtronic, Minneapolis, MN, USA) with an anti-adhesive barrier was selected. The inferior defect was closed with a V-Loc 180 barbed suture (Covidien, Mansfield, MA, USA) and reinforced with an 8 × 8 cm composite mesh secured circumferentially with a V-Loc 90 barbed suture. The superior defect was closed primarily with a 2-0 Vicryl (polyglactin) figure-of-eight suture and reinforced with a 6-cm round composite mesh. The peritoneum was reapproximated to the lateral abdominal wall. Musculofascial closure of all trocar sites was performed using 0 Vicryl (polyglactin) sutures placed with a suture passer.

**Figure 5:**
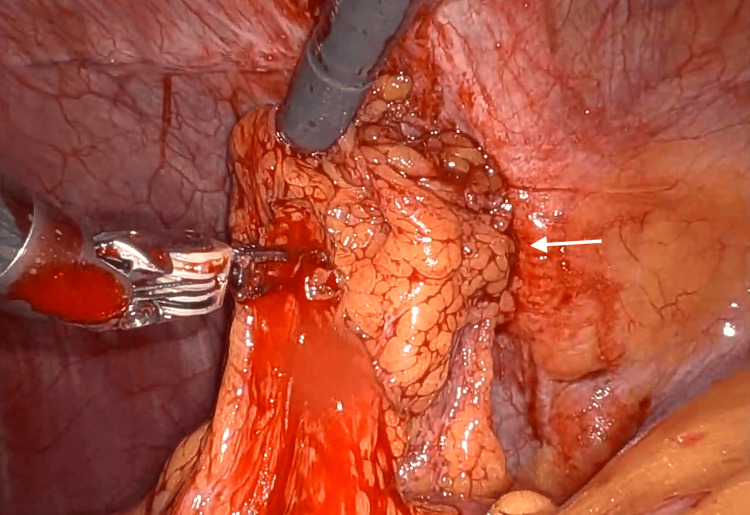
Robot-assisted dissection of incarcerated omentum from the left inferior trocar-site hernia sac

Following robot-assisted completion of the mesh repair, the left inferior trocar site was opened to resect the remaining incarcerated omentum and hernia sac using electrocautery. The abdominal wall was closed in layers: internal oblique with 2-0 Vicryl (polyglactin), external oblique and fascia with 2-0 Vicryl (polyglactin), and Scarpa's fascia with 3-0 Vicryl (polyglactin). Skin was closed with subcuticular absorbable suture. Estimated blood loss was minimal. The patient tolerated the procedure well and was discharged the same day. 

At subsequent follow-up, the patient's recovery was uncomplicated with no evidence of hernia recurrence.

## Discussion

This case illustrates a rare complication of robot-assisted incisional hernia repair. Simultaneous TSHs were identified at three 8-mm port sites within two weeks of the index operation. The occurrence of multiple TSHs in a single patient following robot-assisted surgery has not been well described. 

Most reported TSHs involve a single site, typically at a 12-mm port or an extraction site, and present months to years after surgery. In contrast, this patient developed hernias at all three 8-mm trocar sites within two weeks, with one incarcerated at the time of reoperation. This timeline suggests that fascial failure may have begun in the immediate postoperative period. 

Evidence on TSH following robot-assisted surgery remains limited. The largest prospective study of 8-mm trocar sites reported a rate of only 0.2% at a median follow-up of 14.5 months [7]. A retrospective review of 178 patients undergoing robot-assisted general surgery reported three port-site hernias at 8-mm sites out of 433 ports (0.7%), with two requiring emergent repair for small bowel incarceration; however, none of these patients developed hernias at multiple port sites simultaneously [10]. However, a long-term study with a minimum five-year follow-up reported an 18% TSH rate following robot-assisted inguinal hernia repair, though the majority of these hernias occurred at midline trocar sites rather than at lateral 8-mm ports [9]. In the present case, three TSHs developed at 8-mm sites in the early postoperative period, indicating that the risk extends beyond midline port sites and delayed presentation. 

The patient’s history of recurrent herniation and the presence of diastasis recti likely contributed to this outcome. Diastasis recti has recently been associated with increased risk of trocar-site herniation following robot-assisted surgery, and the pattern of herniation at every trocar site raises the possibility of a generalized susceptibility to fascial failure, characterized by altered collagen metabolism, which has been implicated in the pathogenesis of both incisional hernia and diastasis recti [11-13]. Specific data on the patient's postoperative physical activity were not available. The patient was counseled on standard activity restrictions, including a 20-pound lifting restriction. The simultaneous development of hernias at all three trocar sites argues against activity-related strain as the primary etiology and instead suggests a constitutional weakness of the abdominal wall. As current European Hernia Society/Americas Hernia Society (EHS/AHS) guidelines do not specifically address 8-mm robotic ports, this case suggests that trocar size alone may be an insufficient criterion for determining the need for closure, and that patient-specific risk factors should also be considered [3]. 

These findings suggest that prophylactic fascial closure of 8-mm trocar sites should be considered in patients with risk factors for fascial failure, particularly those with recurrent hernias or diastasis recti. Vigilant postoperative surveillance is equally important, as this case demonstrates that TSH may present early and progress rapidly to incarceration.

## Conclusions

We present a rare case of three simultaneous TSHs occurring at all 8-mm robotic trocar sites within two weeks following robot-assisted incisional hernia repair, with rapid progression to omental incarceration at one site. This case demonstrates that trocar size alone may be an insufficient criterion for determining the need for fascial closure and that patient-specific risk factors, including recurrent hernias, diastasis recti, and a history of prior laparoscopic procedures, should inform the decision to perform prophylactic closure. Further research is needed to establish evidence-based guidelines for fascial closure of 8-mm robotic port sites and to identify patients who may benefit from routine closure of all trocar sites.
